# Engineering microbial technologies for environmental sustainability: choices to make

**DOI:** 10.1111/1751-7915.13986

**Published:** 2021-12-07

**Authors:** Willy Verstraete, Keren Yanuka‐Golub, Nele Driesen, Jo De Vrieze

**Affiliations:** ^1^ Center for Microbial Ecology and Technology (CMET) Faculty of Bioscience Engineering Ghent University Coupure Links 653 Gent B‐9000 Belgium; ^2^ Avecom NV Industrieweg 122P Wondelgem 9032 Belgium; ^3^ The Institute of Applied Research The Galilee Society P.O. Box 437 Shefa‐Amr Israel

## Abstract

Microbial technologies have provided solutions to key challenges in our daily lives for over a century. In the debate about the ongoing climate change and the need for planetary sustainability, microbial ecology and microbial technologies are rarely considered. Nonetheless, they can bring forward vital solutions to decrease and even prevent long‐term effects of climate change. The key to the success of microbial technologies is an effective, target‐oriented microbiome management. Here, we highlight how microbial technologies can play a key role in both natural, i.e. soils and aquatic ecosystems, and semi‐natural or even entirely human‐made, engineered ecosystems, e.g. (waste) water treatment and bodily systems. First, we set forward fundamental guidelines for effective soil microbial resource management, especially with respect to nutrient loss and greenhouse gas abatement. Next, we focus on closing the water circle, integrating resource recovery. We also address the essential interaction of the human and animal host with their respective microbiomes. Finally, we set forward some key future potentials, such as microbial protein and the need to overcome microphobia for microbial products and services. Overall, we conclude that by relying on the wisdom of the past, we can tackle the challenges of our current era through microbial technologies.

## Graphical abstract

Soil ecosystem services that are directly tied to microbial communities and that can be stimulated and engineered to reach the Sustainable Development Goals. Ecosystem services have been estimated to represent an economic value which equals, in the year 2021, at least up to 50% of the global gross domestic product.

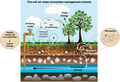

## Introduction

There is an ongoing debate on the concept and urgency of planetary sustainability, which, amongst others, resulted in the identification of the so‐called planetary boundaries (Steffen *et al*., 2015), a conceptual approach that identifies and quantifies human impact on our natural ecosystems. The discussion about the actions that are to be taken for planetary sustainability, however, rarely deal with issues related to microbiology, microbial ecology and microbial technology. Nonetheless, microorganisms play a key role in the Earth’s biogeochemical cycles (Madsen, 2011; Rousk and Bengtson, 2014) and, directly or indirectly, impact the unprecedented climate change that we have to face in the coming decades.

A key feature towards the protection of Earth’s natural resources is that both scientists and engineers must come to more effective management of microbiomes in soils, aqueous environments, but also in relation to bodily systems, such as the rumen, the gastro‐intestinal tract, the skin and even the physiological functions in the human body. For example, soils can act either as carbon source or as sink, and this relates with the activity of soil microorganisms, although knowledge on the quantitative contribution of different phylogenetic groups remains elusive (Gougoulias *et al*., 2014). To achieve proper microbiome management, key generic ecological principles, such as microbial economics (Tasoff *et al*., 2015), the Pareto distribution concept (Dejonghe *et al*., 2001; Verstraete *et al*., 2007; De Vrieze *et al*., 2017), top predator involvements (Chen *et al*., 2011), biostability boundary conditions (Prest *et al*., 2016; Favere *et al*., 2021) and reliable stochasticity (Zhou *et al*., 2014; De Vrieze *et al*., 2020) need to be better defined and taken into account.

These concepts enable a microbial evaluation and management of current full‐scale biotechnologies and allow microbiologists and microbial ecologists to take leadership to speak out on which biotechnology practices should be downscaled and which deserve to be fully promoted. Growing crops that require lots of mineral nitrogen, conventional activated sludge for wastewater treatment, or using ruminants to convert fibrous biomass into edible protein are just three examples that reflect a nutrient‐ and/or energy‐inefficient approach and that require either upgrading or replacement by more resource‐efficient approaches. In contrast, direct and full conversion of wastewater into drinking water, anaerobic digestion, microbial protein production and the use of probiotics are strategies that make the most of the versatility and inherent resource efficiency of microorganisms. A major issue with respect to microbial products and services is that both the consumer and the regulator need to be convinced about the safety and quality assurance of products from natural consortia of microorganisms (De Vrieze *et al*., 2020). Experience from the past in terms of health regulations for microbial products, such as probiotics and bacterial vaccines, particularly live attenuated vaccines, should provide an appropriate framework to foster practices towards microbial products with a well‐balanced evaluation of economic and human health‐related constrains. Overall, microbial biotechnology will be of critical importance in the coming decades and will be at the forefront in our mission of combatting climate change.

Here, we highlight potential key roles of microbial technologies not only in well‐known natural ecosystems, i.e. soils and aquatic ecosystems, but also in semi‐natural or entirely human‐made, engineered ecosystems, e.g. (waste)water treatment and bodily systems, which are, at least partly, based on fundamental microbial ecology concepts. Next, we illustrate how the knowledge gathered in these areas can help guide the world towards a more sustainable, circular future. We further demonstrate through the case of microbial protein how microphobia should be overcome, as this is an underestimated aspect that could severely hinder the future of several important microbial technologies, especially with respect to climate change abatement.

## Effective abilities of mixed microbial communities for engineering our direct environment

When dealing with natural microbial communities, one should consider the concept that not only humans but also microorganisms implement a certain form of market economy (Werner *et al*., 2014). Microorganisms have been shown to participate in collaborative behaviour, both with other (micro)organisms and with the host (Werner *et al*., 2014) of which the human (and virtually all (in)vertebrates) gut ecosystem can be considered a key example (Hooper *et al*., 2002; Lee and Hase, 2014). The similarity between economics and microbial behaviour even goes further, as trading of essential resources between microorganisms can accelerate the total growth of the microbial community (Tasoff *et al*., 2015). In an open system, in which immigration of new microorganism can influence microbial assembly and function in the receiving engineered or natural ecosystem (Mei and Liu, 2019; Dottorini *et al*., 2021), the complete process is carried out with more accuracy and a higher efficiency by multiple species than on an individual basis, as for example illustrated in anode biofilm assembly in microbial fuel cells (Yanuka‐Golub *et al*., 2021).

Overall, using mixed microbial communities allow achieving more effective and efficient microbial processes, because the most suitable species/community combinations will be selected and become operational. Microbial biotechnologists, particularly in the framework of reaching the United Nations Sustainable Development Goals, need to explore with great priority how to optimize and manage such cooperative communities, i.e. the microbiomes as they occur in soils, aquatic environments, engineered reactor systems and even in bodily systems. To this end, it is our role as scientists, to develop a set of pragmatic tools/answers, allowing practitioners and policymakers to select the most appropriate microbial biotechnology. An example of such a scheme is rewilding with invertebrates and microorganisms to restore ecosystems (Contos *et al*., 2021).

### Fundamental guidelines for soil microbial resource management

#### The issues and potentials in soil ecosystems

**Fig. 1:**
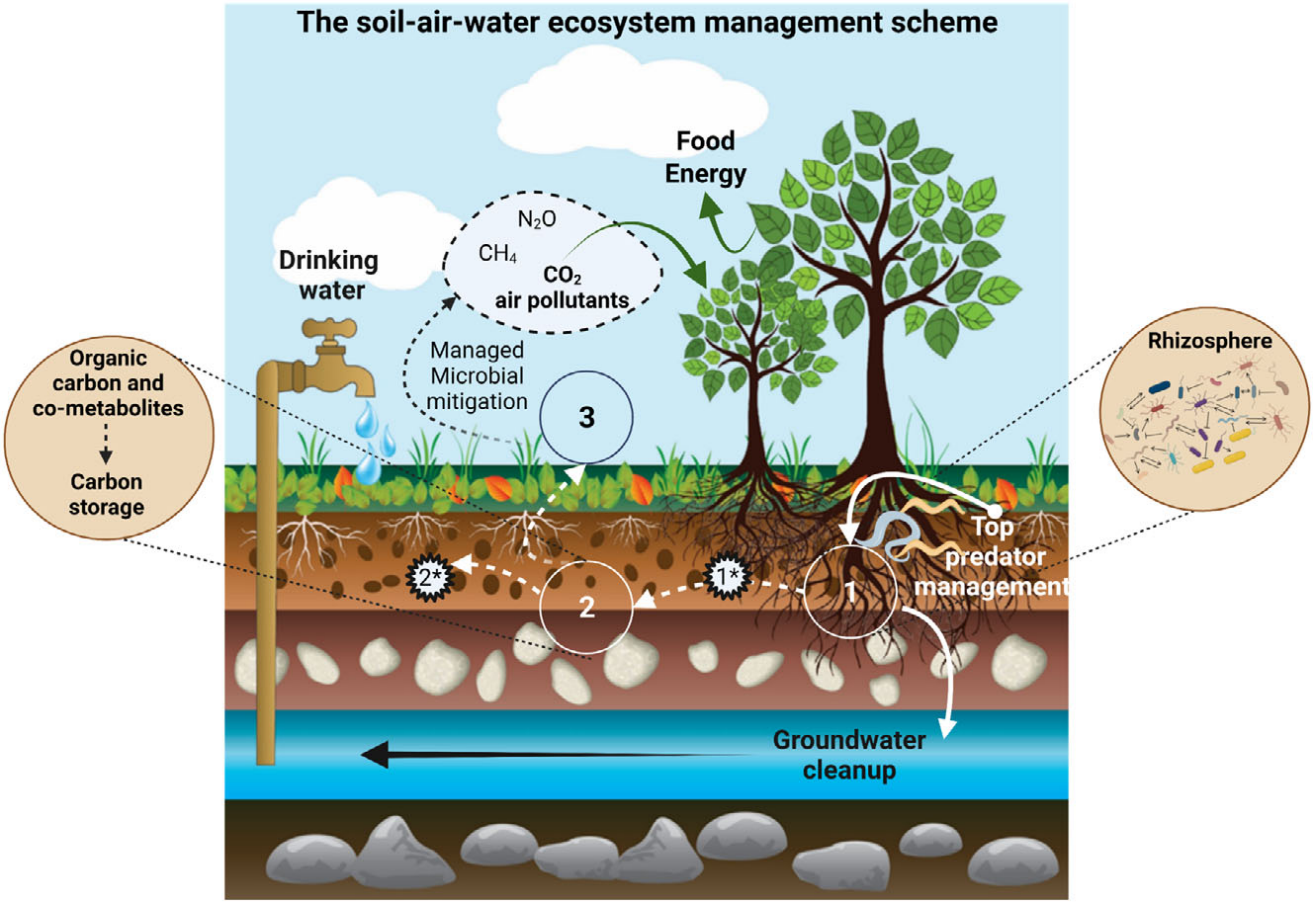
Soil ecosystem services that are directly tied to microbial communities and that can be stimulated and engineered to reach the Sustainable Development Goals. (1) The rhizosphere in which intimate plant–microorganisms interactions, e.g. driven by the management of top‐predators of the microorganisms, can improve plant growth for purposes such as food and feed, energy crops and groundwater production. (2) Organic and inorganic matter produced by plants (indicated as 1*) and microorganisms, e.g. in grasslands, thus, contributing necromass formation and carbon storage (indicated as 2*). (3) Proper soil microbial management can reduce greenhouse gas concentrations in the atmosphere by removal of CO_2_, CH_4_, N_2_O and other pollutants from the air.

There is a tremendous amount of knowledge about soil microbiology, but strategies that are actively modifying the soil microbial community or steering it towards a particular performance are lacking. Several soil ecosystem services, such as exchange of the greenhouse gases CO_2_, CH_4_, and NOx, are well documented (Lal, 2004) and highly depend on microbial activity and their interaction with the local soil environment and land‐use (Galbally *et al*., 2010; Oertel *et al*., 2016). Soil ecosystems contribute substantially to ecosystem services through e.g. upholding rainwater, purifying contaminants from groundwater, and stimulating plant growth for energy and crops through proper nutrient delivery (Fig. 1). These ecosystem services have been estimated to represent an economic value that equals, in 2021, at least up to 50% of the global gross domestic product (Costanza *et al*., 1997, 2017). When translated to a current global human population of 7.9 billion (www.worldometers.info/world‐population/), ecosystem services represent a monetary value of about €5,000 per person per year (Statista, 2021). Hence, we ought to ask ourselves about the benchmarks and the tools needed to preserve these services and even to further increase them. We need to study to what extent the microbial community in soil ecosystems can be enhanced without the risk of opposite, undesirable results.

The need to control anthropogenic greenhouse gas emissions is apparent, but we should also consider the fact that natural ecosystems emit an amount of CO_2_ that roughly equals anthropogenic CO_2_ emissions (Yue and Gao, 2018). The key difference lies in the fact that natural greenhouse gas emissions and sinks balance each other out, whilst anthropogenic emissions result in a net increase in greenhouse gases in the atmosphere. Proper agricultural soil management allows us to achieve carbon sequestration in the range of 0.3‐1.0 tonne C per hectare per year (Denman *et al*., 2007). Hence, if we would consider all agricultural soil in the world to be used as grassland, i.e. about 4.8 billion hectares in 2018, and a global anthropogenic greenhouse gas emission of 36.2 Gton CO_2_ equivalents in 2016 (Yue and Gao, 2018), it should be possible, at a sequestration rate of 1.0 tonne C per hectare per year, to sequestrate up to 50% of all anthropogenic greenhouse gas emissions. However, a more conservative approach estimates a carbon sequestration potential in cropland soils of 5‐20% of all anthropogenic greenhouse gas emissions (Zomer *et al*., 2017). Even though in practice, this is a challenging approach, it demonstrates that appropriate agricultural soil management has a huge potential in terms of greenhouse gas emission abatement.

Turning to one of the other top priority concerns of the planet, i.e. the excessive release of reactive nitrogen fertilizer in the soil (Steffen *et al*., 2015; Seitzinger and Phillips, 2017), it becomes apparent that soil nitrogen management is highly inefficient. About 60% of applied fertilizer N gets lost and, at least partially, ends up in the receiving water bodies, where it induces eutrophication (Vlek and Byrnes, 1986; Isermann, 1990; Cassman *et al*., 1998). This huge loss of nitrogen seems directly related to non‐managed microbial processes in the agricultural ecosystems, such as nitrification and denitrification. Hence, there is an obvious need to better deal with these microbial‐based inefficiencies. In regions with high livestock intensity farming, e.g. in Flanders and the Netherlands, the emission of ammonia and the subsequent deposition in nearby natural ecosystems have become of critical concern because the deposition of reactive nitrogen, amongst others, decreases plant diversity (Erisman *et al*., 2014). This has, for the first time in history, led to the implementation of unprecedented and harsh legislative restrictions on ammonia and nitrate emissions in agriculture by these regions. Such measures are essential because depositions of several tens of kg of ammonia per ha per year in natural ecosystems do affect plant ecology, soil microbiology and the concomitant ecosystem services.

#### Managing the soil microbiome

To manage open microbial communities, there is a need to comprehend how they function. Fortunately, we can once more rely on analogies between ecological and economic systems. As the Italian economist Pareto described in 1897 (Pareto, 1897), in open competitive environments, selection and competition results in a 20/80 distribution pattern, i.e. 20% of the population gets 80% of the flux of goods. However, the other 80% of the population is striving to become more effective, thus, constantly sustains the push for better performance of the overall population. This concept also applies to microbial communities (Marzorati *et al*., 2008). In soils, when measuring the relative/absolute abundance of taxa relative to their role in the bioconversion process, often a Pareto distribution can be observed, even though the 20/80 distribution is not always entirely met (Marzorati *et al*., 2010). This also applies to engineered ecosystems, such as anaerobic digestion (De Vrieze *et al*., 2018). Hence, an evaluation can be made about the overall ecology, vitality and association effectiveness of the microbial community by means of this parameter, which integrates much more information than the listing of species that are present or absent (Verstraete and Mertens, 2004).

Another important concept is the role of the top predator in keeping populations healthy and divers (Thakur and Geisen, 2019). In the soil ecosystem, nematodes are predating on microorganisms (e.g. bacteria and archaea) and thus, support biomass turnover and the release of nutrients (Gebremikael *et al*., 2016). The extreme spatial and temporal heterogeneity of soil gives rise to a wide range of different surface types, aggregate, pore sizes and microclimates, allowing habitat partitioning in space and time by different soil fauna (Stockdale *et al*., 2019). The fact that soil microbial processes are rarely linked to the overall micro‐mesobiota configurations is inadequate, leading to an incomplete picture of system function. Soil microbiology needs to be examined in the context of food chains, including interspecies metabolic interactions, and more efforts to engineer the broader biota, inclusive the top predator(s) are to be developed. For example, the characterization of fatty acid profiles of nematodes and fungi could be used for the detection of specific tropic interactions in the soil (Ruess *et al*., 2002).

The direct interaction between the soil microbiome and higher organisms, e.g. plants, could also be an important aspect towards soil ecosystem management for several purposes. A higher plant diversity accelerates carbon sequestration in degraded and abandoned agricultural soils (Yang *et al*., 2019). The intimate relationship between microorganisms and plants in the rhizosphere and phyllosphere of these plants can be successfully harnessed for the removal of soil and air contaminants (Ramos *et al*., 2009). Recently, it has been shown that by imposing weak electric fields on contaminated soils, electro‐kinetic technologies facilitate improved activity of plant‐associated microorganisms and enhance hydrocarbon pollutants degradation in the soil (Huang *et al*., 2019).

Another element of importance is the recently recognized value of microbial necromass. The formation of such slowly decomposing organic matter in the soil is strongly dependent on the type of plant (crop) present, the soil, and also the type of fertilizer applied (Liang *et al*., 2019; Buckeridge *et al*., 2020). The return of appreciation for organic fertilizer can be considered a positive trend in terms of soil fertility and diversity (Herencia *et al*., 2007; Zhong *et al*., 2010). Hence, such an approach should be promoted and facilitated, e.g. through new pathways to produce microbial biomass, such as microbial protein (Pikaar *et al*., 2018c), to be used to generate necromass in the soil.

#### Employing the soil microbiome for greenhouse gas abatement

**Fig. 2:**
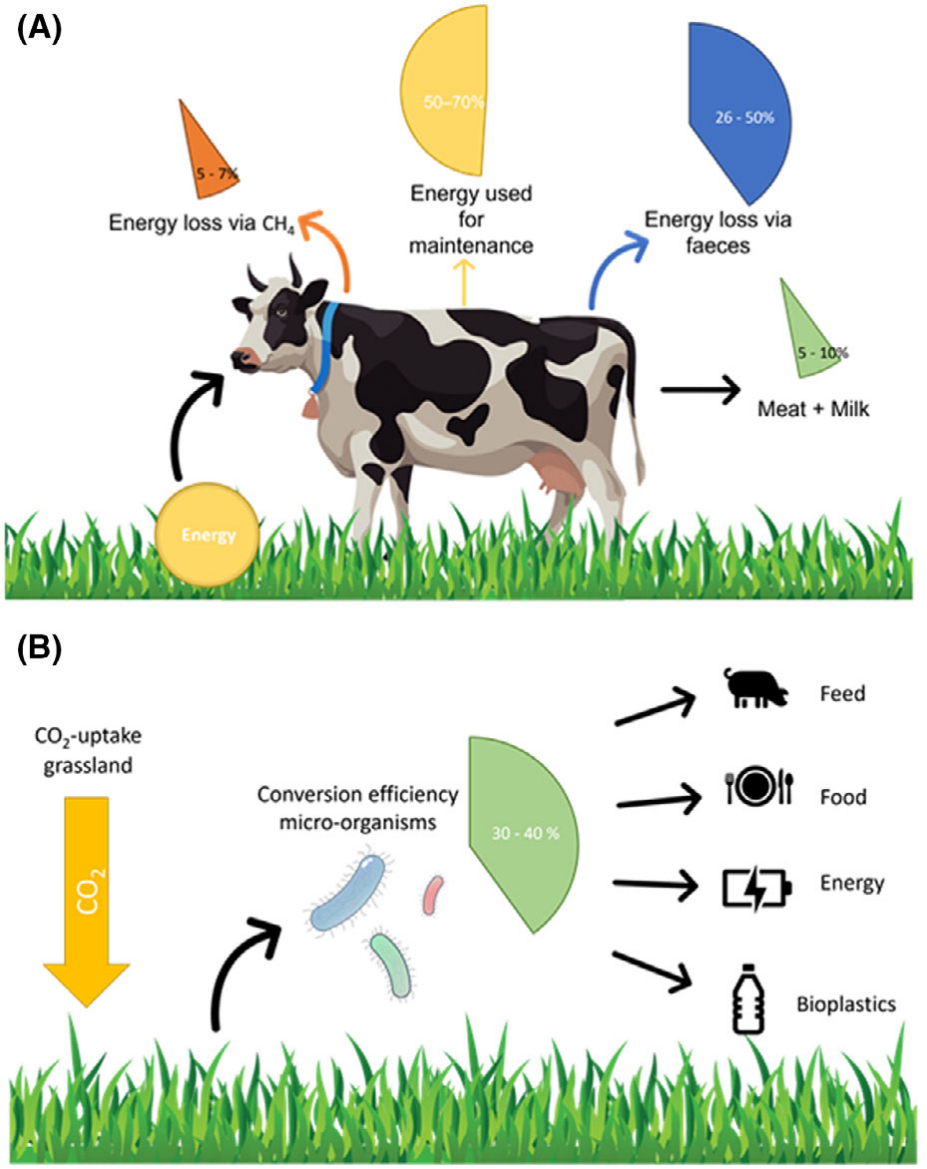
Comparison between the resource‐inefficient ruminant‐route (A) versus the more efficient, valorisation of grass cellulose fibres, directly through microorganisms within the context of the biorefinery (B).

**Fig. 3:**
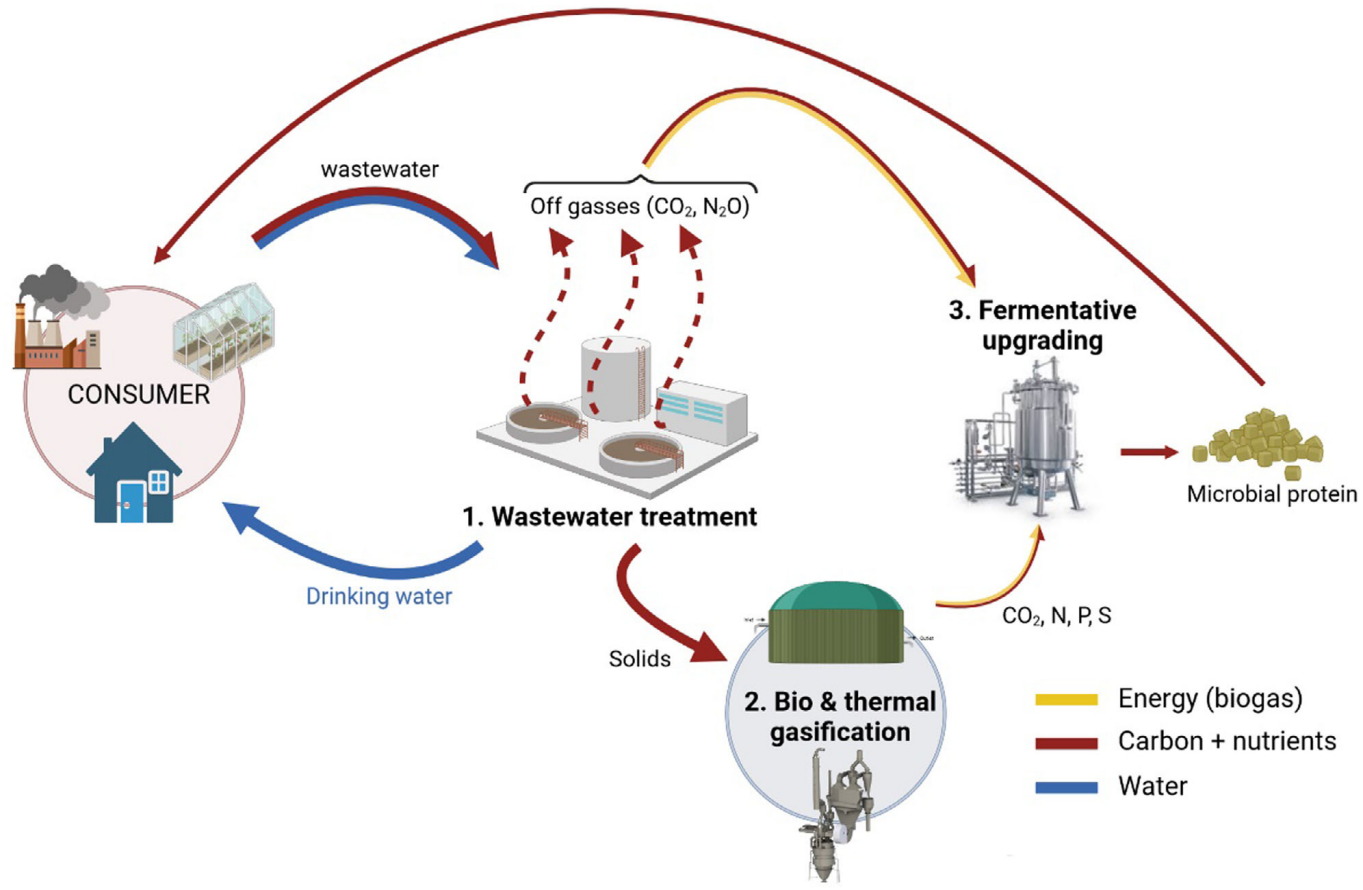
Schematic overview of the circular biorefinery concept. Wastewater is biologically treated at a wastewater treatment plant (1), during which microbial biomass is produced, thereby capturing CO_2_ and adsorbing the bulk of the contaminants in and onto their biomass. The microbial biomass (solids) is subsequently processed by biological (anaerobic digestion) and thermal gasification (2) to clean and re‐usable gases. Thus, the infinite variety of molecules (including recalcitrant pharmaceuticals) can be rendered to become reliable, conceptually safe and re‐usable gaseous components. These gases are used in a second aerobic fermentation process (3) to produce clean microbial biomass. This closes the resource‐bio‐recovery loop and produces microbial biomass that is fit to be used as food, feed, organic fertilizer or bio‐polymer, thereby returning back to the consumer.

Soil scientists can contribute in the framework of the CO_2_ abatement in several ways. First, as demonstrated in the Underground Sun Storage project in Austria (Pichler, 2019), deep soils, previously holding natural gas, can be used as reservoir for the production and storage of renewable methane. This methane is produced *in situ* by the activity of hydrogenotrophic methanogens, using renewably produced hydrogen gas and CO_2_ from methane burning in a cyclic process. Second, soil scientists should engage in the promotion of the biorefinery for the production of carbohydrate‐rich biomass as a product in itself. Perennial grasslands, for example, not only produce grass biomass without major inputs of fertilizer but also can store (as indicated above) about one tonne of organic carbon per ha and per year. Such carbohydrate‐rich biomass is, at present, consumed to large extent by ruminants. The overall efficiency of the ruminant is, however, quite low. Ruminants digest 60‐70% of grass fibre, and of the digested part, about 15% is converted to products, such as meat or milk (Buxton and Redfearn, 1997; Britt *et al*., 2003). Hence, expressed on a dry matter basis, hay yields about 0.10 kg of milk dry matter per kg dry matter fibre (Fig. 2). The organization of a biorefinery, as depicted in Fig. 3, in which the carbon‐ and nitrogen‐rich components of the crop, e.g. grass (Kamm *et al*., 2016), are microbiologically ‘upgraded’ to generate feed, food and commodities, such as fatty acids (Jones *et al*., 2021), and to recover energy (biomethane from biogas) deserves strong promotion. Such a biorefinery can bring harmony between (i) the soil ecosystem and its services (ii) and the agricultural logistics and economics.

### Closing the water circle: wastewater reclamation and resource recovery

#### The need for direct potable re‐use of reclaimed water

Nothing is more precious than top‐quality drinking water. Given our intense use of water, both for domestic and industrial applications, water scientists should have the ambition to valorise used water to, e.g. drinking water or process water in a fit‐for‐purpose approach (Muller, 2010; Capodaglio, 2021). Currently, we indirectly re‐use substantial amounts of surface water discharged by wastewater treatment facilities upstream of the drinking water production site. The need to directly use ‘reclaimed water’, i.e. effluents from wastewater treatment plants, will strongly increase in the coming decades, particularly during periods of drought (Leverenz *et al*., 2011). An excellent example of direct potable re‐use of purified wastewater can be found in Koksijde, Belgium, where since early 2000, drinking water delivered to the consumer is based on 50% reclaimed water (Van Houtte and Verbauwhede, 2008) and even aquifer recharge is achieved (Van Houtte and Verbauwhede, 2021). Clearly, the advanced removal of minor pollutants by systems, such as slow sand filtration, are well established (Sharma and Bhattacharya, 2017), and the additional questions about the biostability of the water produced can be handled. Engineers can characterize biostability by considering the presence of nutrients relative to the presence of a sufficient and diverse microbial community (Favere *et al*., 2021).

#### Evolving beyond conventional activated sludge

The activated sludge system is often considered the ‘pinnacle’ of microbial biotechnology, and its origin dates back more than a century (Ardern and Lockett, 1914). The microorganisms in the activated sludge ecosystem display an excellent example of microbial collaboration, by which polluted ‘stinky’ wastewater ‘disappears’. However, we should dare to speak out on whether this technology is appropriate and still relevant for the coming decades. In the present era of resource recovery, amongst others, from municipal wastewater (Verstraete *et al*., 2016; Puyol *et al*., 2017), the conventional activated sludge system displays some key disadvantages. Conventional activated sludge systems (i) consume a lot of energy, (ii) remove and do not recover nitrogen in the form of nitrogen gas, and also for several percentages emit N_2_O, which has an enormous global warming potential, and (iii) rarely are discharging water at sufficient quality for (in)direct re‐use (Verstraete and Vlaeminck, 2011; Sheik *et al*., 2014). Some conventional activated sludge plants include anaerobic digestion for energy recovery, but energy conversion efficiencies for waste activated sludge to biogas remain in the order of only 21–36% at mesophilic conditions (De Vrieze *et al*., 2016). Others recover phosphorus (Sørensen *et al*., 2015) or other commodities (Puyol *et al*., 2017), but the majority is simply built and operated to make sewage dissipate. The revalorisation of the Adsorptions‐Belebungsverfahren or AB process (Böhnke, 1977) reflects an excellent approach that enables the conservation of energy contained within the wastewater in the biodegradable A‐sludge, with potential for additional resource recovery (De Vrieze *et al*., 2016; Meerburg *et al*., 2016). Microbial biotechnologists should dare to reconsider this way of using microorganisms. A new concept that was recently proposed for sustaining biological wastewater treatment is the ‘microbial niche nexus’ (Wu and Yin, 2020). Through tuning microbial niches to accommodate diverse microbial communities and unique microenvironments, this concept could be applied to solve emerging challenges by considering true microbial ecology concepts, e.g. r/K‐strategists (Pianka, 1970; De Vrieze *et al*., 2017), besides infrastructure issues. Overall, such an approach lays the foundation to progress towards a more holistic approach in developing biotechnological solutions for wastewater treatment.

#### Anaerobic digestion: an old technology with future potentials

Anaerobic digestion can be considered a marvellous biotechnological process that has the potential to grow even further in importance in our future bio‐based circular economy (Acosta and De Vrieze, 2018; Wainaina *et al*., 2020). The fact that a consortium of microorganisms synergistically breaks down a multitude of complex molecules, and then ultimately generates biogas, composed mainly of CH_4_ and CO_2_, representing about 90% or even more of the (biodegradable) energy input, is astonishing in its overall thermodynamics, biochemistry and applicability. Plenty of technological configurations, ranging from the upflow anaerobic sludge blanket reactor systems (Lettinga *et al*., 1980) to solid‐state digesters (Six and De Baere, 1992) have been invented and developed into full‐scale applications over the years, and they render tremendous services to society. The emergence of high‐throughput molecular methods in the last decades greatly accelerated our insights into the microbial ecology of anaerobic digestion (Vanwonterghem *et al*., 2014; Cabezas *et al*., 2015; Carballa *et al*., 2015). However, it should be acknowledged that up to this day and to the best of our knowledge, not a single synthetic microbiome capable to deal with the ‘entropy’ of waste has been constructed and brought to a performance outranking that of natural microbiomes in anaerobic digestion. Hence, there are still substantial potentials for improvement in terms of rate and residual metabolites to be addressed in the near future.

#### The biofloc: an example of circularity from faeces to food in aqueous ecosystems

**Fig. 4:**
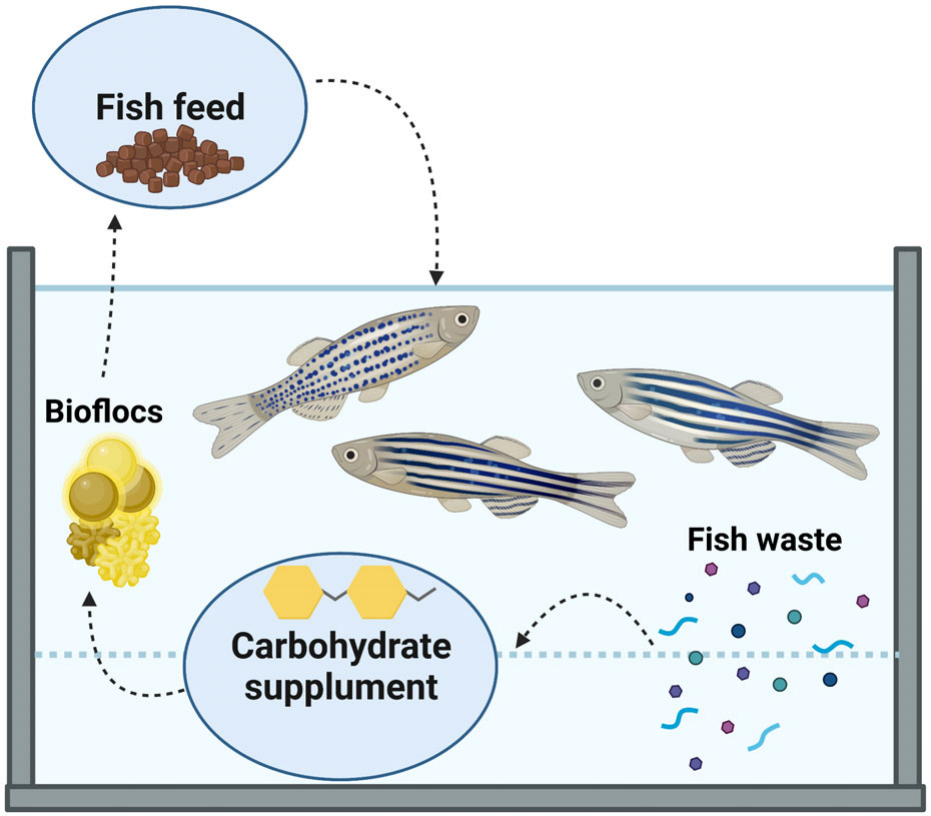
Example of wastewater reclamation and resource recovery to obtain a circular water‐management system in the aquaculture sector. By providing additional carbohydrates, waste produced by fish is converted *in situ* to nutritious microbial biomass, growing in flocs, which can be re‐used again as feed for the fish.

A quite special achievement in microbial biotechnology is the biofloc technology (Crab *et al*., 2012). Some decades ago, at the onset of intensive aquaculture, it became clear that protein‐rich fishmeal implemented was hardly converted to the targeted biomass (e.g. fish, shrimp), and that 20–80% was wasted as faecal matter. By providing additional carbohydrates, it was possible to convert *in situ* the waste products of the aquaculture target organisms to nutritious microbial biomass (Avnimelech, 1999). This biomass would grow in flocs, which could serve as feed for the subsequent or even the same aquaculture production animal (De Schryver *et al*., 2008). This allowed to double the feed conversion efficiency of intense aquaculture systems, whilst cleaning up the otherwise major discharge of wastes. Essentially, this example shows vividly how the circular economy concept, e.g. upgrading faecal matter into food, can be rather easily applied and accepted by the consumer (Fig. 4).

### Bodily systems

The COVID‐19 pandemic has made it evident once again that microorganisms are everywhere in and around us. Since we cannot exclude or even live without them, we should join forces with those that are beneficial to the human body. In that context, a number of considerations in relation to technology come to mind.

A very predominant reactor‐like system is the rumen. It has been well studied, since it effectively converts cellulose fibres to higher products, such as meat and milk. This established fact, however, should be judged in relation to the environment. As mentioned before, ruminants are not very effective in converting cellulose fibres to milk or meat, with an overall feed energy conversion efficiency to protein (milk or meat) of only 10%. In addition, the rumen converts 5–7% of energy in the feedstock into the greenhouse gas methane, whilst 26–50% of the energy is lost via the faeces (Arndt *et al*., 2015) (Fig. 2). The emission of methane by cows stands for a contribution of 1–2% to the total greenhouse load worldwide (Yusuf, 2012). Several attempts have been made to reduce the emissions of CH_4_ by ruminants, such as the addition of polyunsaturated fatty acids, aromatic herbs or specific chemicals, like 3‐nitrooxypropanol, to the feed (Beauchemin *et al*., 2008; Romero‐Perez *et al*., 2014; Valli, 2020). Even though these strategies reduced methane emissions, this was only in the range of 5–10% of the amount produced. Methane production through methanogenic archaea could even be essential to sustain cellulose hydrolysis in the rumen because removing gas and moderating pH could be crucial in biofilm maintenance in the rumen (Mason and Stuckey, 2016). Hence, it appears that the agricultural focus on ruminants is not compatible with a sustainable climate for the future. In that perspective, the way forward is to face the fact that animal husbandry and focus on fibre conversion by ruminants must be downscaled and that upgrading of carbohydrate‐rich fibres is much more directed to specific technologies in a highly controlled biorefinery context (Fig. 3) (Kamm *et al*., 2016).

Over the last decades, microbiologists and microbial ecologists have discovered and explored the human gastro‐intestinal microbiome, both in vivo and in powerful simulators, such as the Simulator of the Human Intestinal Microbial Ecosystem (SHIME^®^) (Van de Wiele *et al*., 2015). Molecular techniques have allowed to detect and identify a tremendous wealth of microorganisms thriving within us and clearly influencing not only our energy balance, uptake of amino acids, minerals and vitamins but also our immunology and even our psychological well‐being (Pennisi, 2019; Lee *et al*., 2020). Thus far, the major emphasis has been on analysis, but time has come for individual management of our fragile and complex gut microbiome, as for example illustrated in the link between the human gut microbiome and mental health (Valles‐Colomer *et al*., 2019).

A final aspect of bodily systems relates to the microorganisms around us that we encounter on daily basis. There have been several indications that overall diversity, including microbial diversity, around us reduces the appearance of allergies (Lambrecht and Hammad, 2017; Ruokolainen *et al*., 2017). In addition, there seems to be a connection between the ongoing decline in biodiversity and the increase in chronic inflammatory diseases in humans. This can be translated in the ‘hygiene hypothesis’, which states that environments rich in microbial diversity offer protection against allergic and autoimmune diseases (von Hertzen *et al*., 2011; Lambrecht and Hammad, 2017). However, while we so far maintain our well‐established methods of maximal disinfection of our immediate environment to protect our health, the concept of our ‘old friends’ is worth examining much more in the future (Rook *et al*., 2004). The common issue of healthcare‐associated issues in hospitals could be dealt with by applying a probiotic‐based sanitation, which could decrease surface pathogens up to 90% more than conventional disinfectants, without the risk of selecting for resistant species (Caselli, 2017). Overall, there is great need to engineer the air and the surfaces in e.g. our homes, schools, hospitals. It is imperative we dare to think outside the box with respect to the conventional and certainly COVID‐19 empowered lines of eradication of every microorganism that can reach us.

## Microbial biotechnologies to be promoted in full force: specific aspects

The considerations so far have indicated that there is a multitude of lines of action in our environment that deserve attention. Two aspects deserve special focus, of which one is technical and the other is overarching both the consumer and the regulatory issues.

### Microbial protein revisited: a reconsideration and future perspectives

The established practice of the biofloc technology (De Schryver *et al*., 2008) demonstrates that waste streams (even faecal waste) can be upgraded by a reliable microbial value chain to, for example in the case of the bioflocs, fish protein. The central element is the fact that microbial cells can contain up to 75–80% protein, rich in all essential amino acids and with a high digestibility (Matassa *et al*., 2016; Clauwaert *et al*., 2017). The concept of single‐cell or microbial protein dates back around fifty years ago, with ‘Pruteen’ as microbial protein supplement (Braude *et al*., 1977).

A combination of, amongst others, low prices for soy, particularly in the USA and Brazil, and fishmeal, increased oil prices and a high nucleic acid content (up to 15–16% of the dry cell weight) resulted in the temporal discontinuation (Anupama and Ravindra, 2000; Clauwaert *et al*., 2017). Currently, the further increase of soy cultivation comes to its limits, and also the harvesting of massive amounts of fish in the high seas is no longer an area where further expansion is desirable (Jeremy *et al*., 2001; Coll *et al*., 2008). Therefore, time has come to revisit microbial protein.

Several lines of production of microbial protein can be envisioned, including autotrophic growth with natural sunlight (Capson‐Tojo *et al*., 2020; Leger *et al*., 2021), autotrophic growth with for instance hydrogen gas produced from water electrolysis using renewable energy (Matassa *et al*., 2015b), and heterotrophic growth with organic matter, such as carbohydrates produced from conventional crops (Pikaar *et al*., 2018a). In all cases, the yield of microbial protein is at least a factor three higher in terms of energy input than for higher organisms, except for phototrophic algae (Matassa *et al*., 2015a). Phototrophs can attain carbon conversion efficiencies to biomass in the order of 100%, because they benefit from a light‐driven anabolism, whilst organotrophs can reach a carbon conversion efficiency to biomass in the order of 30–40% because they consume part of the energy contained within the organic carbon sources in aerobic metabolism (Matassa *et al*., 2015a). In contrast, higher organisms, such as insects and other animals reach carbon conversion efficiencies to biomass in the order of only 10%. This difference between prokaryotes and eukaryotes can be explained by the fact that bacteria have no complex organs or cellular components to produce, with a relatively small genome. However, in contrast with higher organisms, the end‐product of microbial protein production is ‘just’ protein, thus far lacking a clear taste and texture.

Nonetheless, microbial protein fits perfectly within the concept of planetary sustainability, based on its energy efficiency and through life cycle analysis (Järviö *et al*., 2021; Khoshnevisan *et al*., 2020; Sillman *et al*., 2020), but it needs further research to detect and promote high‐value ingredients. An essential aspect is that the quality of the input determines the quality of the recovered protein, i.e. the GIGO principle (garbage in, garbage out) also, to a certain extent, applies to microbial protein. Depending on the characteristics of the microbial protein product, it can be used as food, feed or implemented as an organic fertilizer (Pikaar *et al*., 2018c), thus, opening the potential to have more microbial necromass in the soil system or even produce commodities. It can be projected that, by 2050, substituting 10‐19% of plant protein for microbial protein could liberate up to 13% of agricultural soils, which can be used for other purposes (Pikaar *et al*., 2018b). Expanding this replacement and/or directly using microbial protein as food source in the future could free up even more agricultural soils, enabling land owners to shift their focus from food production to other soil management strategies, such as the reduction of greenhouse gas emissions or promoting biodiversity. Recently, it was even demonstrated that microbial protein, produced from starch, could serve as protein source for the production of bioplastics (Singha *et al*., 2021), which reflects a huge market potential. Clearly, the time has come to carefully delineate the future of microbial protein.

An important aspect is that, to generate microbial protein from waste streams (e.g. sewage), either directly (Vethathirri *et al*., 2021) or indirectly over methane (Tsapekos *et al*., 2020; Zha *et al*., 2021), one should face the fact that the organics in these waste streams are most probably contaminated with chemicals, bacteria and viruses, which need to be avoided. By combining anaerobic digestion with thermochemical gasification, organic waste streams can be converted to clean gaseous substrates in the ‘full gas’ route (Matassa *et al*., 2020), which can be used for the safe production of microbial protein through various routes (Fig. 3).

### Overcoming microphobia for microbial products

In the free market economy, it is essential that one touches base with the consumer. The consumer is willing to accept a shift towards a circular bio‐economy, provided the products generated are appealing in terms of price and, especially, safe in terms of use (Maria *et al*., 2015). The aspect of safety of products generated by means of microbial biotechnology is not a minor issue and is further complicated since the consumer demands rigorous protection by the regulator. In the past, multiple products, such as drinking water using slow sand filters, sourdough, cheese using raw milk, Gueuze beer and wine have been produced by means of open, mixed microbial communities, i.e. by allowing the natural selection of microorganisms, some even with specific health benefits (Marco *et al*., 2017). These products are generated under particular conditions of ‘good manufacturing practices’, and a set of analyses targeting indicator organisms, for example non‐tuberculous mycobacteria in drinking water (Dowdell *et al*., 2019), are demanded. The development and implementation of the ‘omics’ in the last decade allows us to detect every species, even present in a nominal, minor concentration, in such open microbiomes. In case the ‘omics’ approach and the total absence of any species/strain not having a positive recognition on the Generally Recognized As Safe (GRAS) list becomes the line of safeguarding by the authorities, then the future applicability of open biotechnology for, amongst others, (waste)water treatment, composting, mushroom production, winemaking, cheese production from raw milk, will become very unpractical and costly. Constructive thinking about operational frameworks to deal with microbiomes have been set forward (De Vrieze *et al*., 2020). It is of crucial importance that the consumer and the regulator are constantly informed about all the aspects of the microbial biotechnology and their respective hazards and control points, so that the risk is kept minimal, whilst realizing that ‘zero risk’ is impossible, and the potential of control and remediation is always at hand. It is imperative to educate the broader population that to strive in an effective way to sustainability, it is wise to cooperate with the set of microbial partners with whom we co‐habit this planet.

## Conclusions

In this paper, we highlighted the potential of using microbial technologies to empower microbial processes with great yet commonly underestimated significance towards planetary sustainability and human health. We emphasized the need to redirect microbial technologies from analytical to systemic thinking in a holistic approach. We argued that the regulator should have confidence in the stochasticity of the microbiomes, and should rely on the wisdom of the past to assure that the potentials of the microbiome and their ecosystem services can further support our planet.

## Acknowledgements

The authors would like to thank Eva Top for her valuable input.

## Conflict of interest

None declared.
